# Association of Serum Uric Acid Level and Thyroid Function in Chronic Kidney Disease: A Hospital-Based Cross-Sectional Study From Northeast India

**DOI:** 10.7759/cureus.69392

**Published:** 2024-09-14

**Authors:** Polina Boruah, Alice Ruram, Arup Jyoti Baruah, Balary Nongtdu, Bhupen Barman, Debashis Priyadarshan Sahoo, Chandan Nath, Jayanta Das

**Affiliations:** 1 Department of Biochemistry, North Eastern Indira Gandhi Regional Institute of Health and Medical Sciences (NEIGRIHMS), Shillong, IND; 2 Department of General Surgery, North Eastern Indira Gandhi Regional Institute of Health and Medical Sciences (NEIGRIHMS), Shillong, IND; 3 Department of Internal Medicine, All India Institute of Medical Sciences, Guwahati, Guwahati, IND; 4 Department of Biochemistry, All India Institute of Medical Sciences, Guwahati, Guwahati, IND

**Keywords:** chronic kidney disease (ckd), creatinine (creat), serum urea, serum uric acid level, thyroid function test (tft)

## Abstract

Background: Although the prevalence of thyroid dysfunction and hyperuricemia are independently high in patients with chronic kidney disease (CKD), there are limited data showing the association of serum uric acid and thyroid function in those with CKD.

Aim and objectives: The aim of this study was to observe the alteration of both the serum uric acid level and thyroid function in CKD patients and to find the association between both.

Materials and methods: This observational cross-sectional study was conducted in a tertiary care hospital over a period of one year in Northeast India. A total of 50 CKD patients were enrolled. Their demographic profiles were studied. Serum urea, creatinine, thyroid-stimulating hormone (TSH), total triiodothyronine (TT3), free triiodothyronine (FT3), total tetraiodothyronine (TT4), and free tetraiodothyronine (FT4) levels were measured to establish the correlation of serum uric acid along with each of the parameters separately. A p-value of *<*0.05 was considered statistically significant.

Results: In the CKD patients studied, serum uric acid exhibited positive correlations with serum creatinine (p = 0.001, r = 0.67), serum urea (p = 0.001, r = 0.69), and serum TSH levels (p = 0.001, r = 0.5). Conversely, serum uric acid showed negative correlations with serum TT4 (p = 0.001, r = -0.74), TT3 (p = 0.001, r = -0.6), FT4 (p = 0.001, r = -0.53), and FT3 (p = 0.001, r = -0.58) levels.

Conclusion: There was a significant positive correlation between uric acid and TSH levels in CKD patients. Thus, early estimation of both parameters should be considered in CKD patients.

## Introduction

Chronic kidney disease (CKD) represents the most common life-threatening consequence of various factors, including metabolic and endocrine disorders, nephritis, hypertension, cardiovascular diseases, and immune system disorders [1]. As these conditions progress, common renal pathological manifestations such as glomerulotubular sclerosis and/or interstitial fibrosis develop, regardless of the underlying causes [2]. In recent decades, advances in cellular and molecular biology have facilitated a better understanding of the pathophysiology of the factors leading to CKD, thereby enabling the development of effective measures to arrest these causes as early as possible to prevent or delay CKD progression [2].

The metabolism and excretion of several thyroid hormones are influenced by kidney function; thus, impaired kidney function is often accompanied by thyroid dysfunction. Consequently, thyroid function derangement is frequently observed in CKD patients [1,2]. However, the overlap in symptoms between the uremic syndrome and hypothyroidism necessitates cautious interpretation of thyroid function tests. Despite this overlap, it is generally possible to accurately assess thyroid function status in each CKD patient through physical diagnosis and thyroid function test. Careful interpretation is essential to differentiate between uremic syndrome and coexisting thyroid dysfunction due to common overlapping symptoms. This differentiation can be achieved by accurately assessing thyroid status via thyroid function tests in CKD patients.

Numerous epidemiological data indicate a positive correlation between predialysis CKD patients and hypothyroidism, with many cases being subclinical [2,3]. Hyperuricaemia may result from either increased uric acid production or decreased renal excretion. In humans, the excretion of uric acid by the kidney involves glomerular filtration, tubular reabsorption, secretion, and post-secretory reabsorption [4,5]. Studying the role of uric acid in CKD is challenging because any impairment of renal function leads to derangement of uric acid levels beyond the normal range. Thyroid hormones also regulate purine metabolism; therefore thyroid disorders may significantly cause abnormal serum uric acid levels.

The present study aimed to observe the association of serum uric acid with thyroid function among CKD patients.

## Materials and methods

This observational cross-sectional study was conducted over 16 months, from November 2022 to February 2024, at North Eastern Indira Gandhi Regional Institute of Health and Medical Sciences (NEIGRIHMS), a tertiary care hospital in Northeast India. The study included 50 CKD patients over 18 years old after obtaining written consent. Ethical clearance was obtained from the Institutional Ethics Committee of NEIGRIHMS before the research commenced (approval no. NEIGR/IEC/M14/T13/2022).

Exclusion criteria included individuals on any hormonal replacement therapy; pregnant women; patients who had undergone total thyroidectomy; those with current or past use of radioiodine and anti-thyroid drugs; individuals with previously diagnosed overt hypothyroidism or hyperthyroidism, thyroid cancer, or thyroid nodules; patients using uricosuric drugs regularly; and those with cardiovascular diseases on antiarrhythmic therapy, such as amiodarone.

Four milliliters of blood were collected into a vacutainer, allowed to clot completely, and then centrifuged at 1000 rpm for 10 minutes at 20°C. The supernatant serum was carefully removed for analysis, ensuring it was free from hemolysis. Serum uric acid, creatinine, and urea levels were measured using uricase, alkaline picrate, and urease methods, respectively, on a fully automated Beckman-Coulter AU5800 analyzer, requiring 450 μL of serum for estimation. Thyroid-stimulating hormone (TSH), TT3 (total 3,5,3'-triiodothyronine), T4 (total 3,5,3',5'-tetra-iodothyronine), FT3 (free 3,5,3'-triiodothyronine), and FT4 (free 3,5,3',5'-tetra-iodothyronine) levels were determined using a chemiluminescent technique on a Beckman-Coulter UniCel DxI 800 Access, with 5 μL of serum required for each estimation with a single standard laboratory reference range (Ref). Residual fibrin and cellular debris were removed prior to analysis.

Before estimating biochemical parameters, the AU5800 instrument was accurately calibrated, and two levels of internal quality controls from Biorad were assessed for each parameter. The results of these controls were accurate according to the Levey-Jennings chart and complied with Westgard rules. Then, external quality control reports from Christian Medical College, Vellore, confirmed the accuracy of the biochemical parameters [6,7].

All data were compiled and analyzed using Microsoft Office Excel 2010 (Microsoft® Corp., Redmond, WA, USA). The association between serum uric acid levels and serum creatinine, urea, and various thyroid parameters (TT4, TT3, FT4, FT3, and TSH) was determined using correlation coefficients. A p-value of <0.05 was considered significant.

## Results

The study enrolled a total of 50 patients, ranging in age from 20 years to 76 years, with a mean age of 46 ± 13.78 years and a male-to-female ratio of 1:1.23. Serum urea, serum creatinine, serum uric acid, TT4, TT3, FT4, FT3, and TSH were estimated in all participants with a mean value of 154.87 ± 54.6 (ref: 8.0-24.0 mg/dL), 8.73 ± 4.87 (ref: 0.7-1.2 mg/dL), 8.1 ± 3.2 (ref: 3.5-7.2 mg/dL), 5.60 ± 2.15 (ref: 5.4-11.5 mg/dL), 40.76 ± 56.34 (ref: 60.0-180.0 ng/dL), 4.23 ± 56 mcg/dL (ref: 5.0-12.0 mcg/dL), and 75 ± 34 ng/dL (ref: 80.0-220.0 ng/dL) respectively. Serum uric acid showed a positive correlation with serum urea (p = 0.001, r = 0.67) and serum creatinine (p = 0.001, r = 0.67).

**Figure 1:**
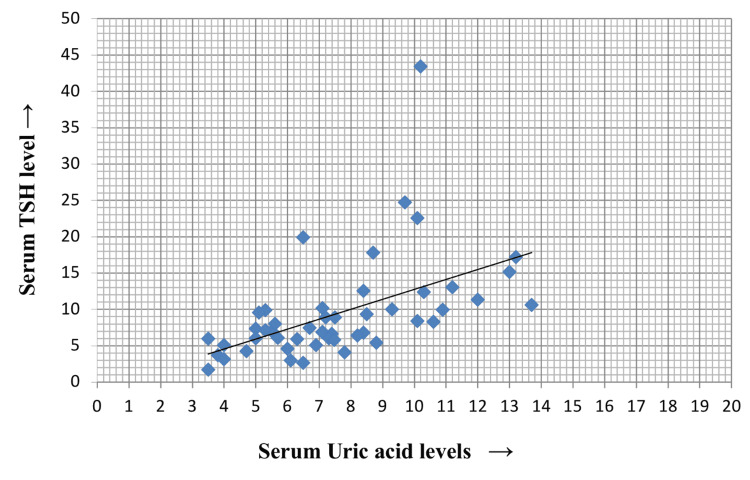
Correlation of uric acid with serum TSH TSH: thyroid-stimulating hormone

Serum uric acid and serum TSH levels were high, and as shown in Figure 1, both the parameters showed positive correlations with each other (p = 0.001, r = 0.5).

**Figure 2:**
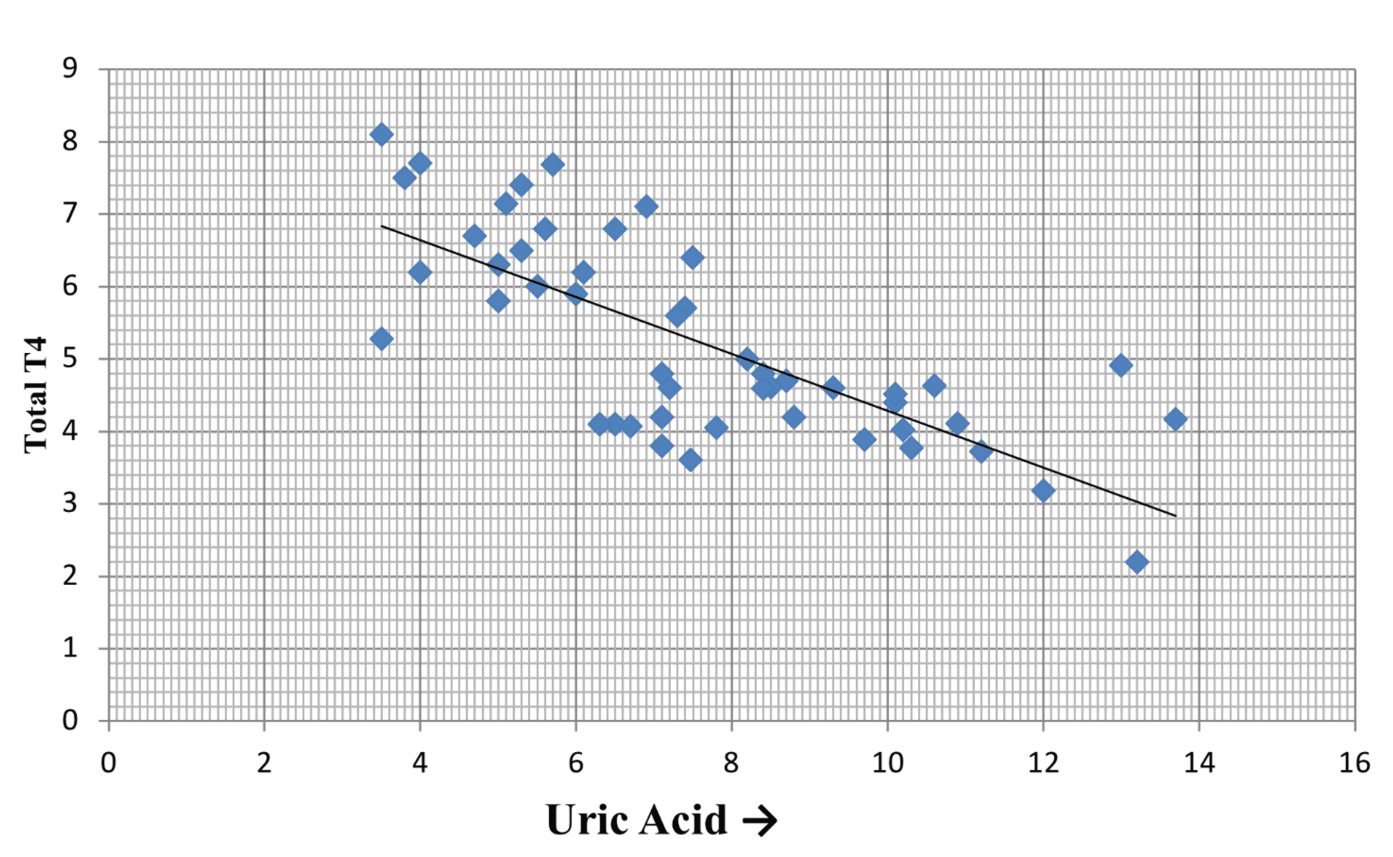
Correlation of serum uric acid with serum TT4 TT4: total T4

**Figure 3:**
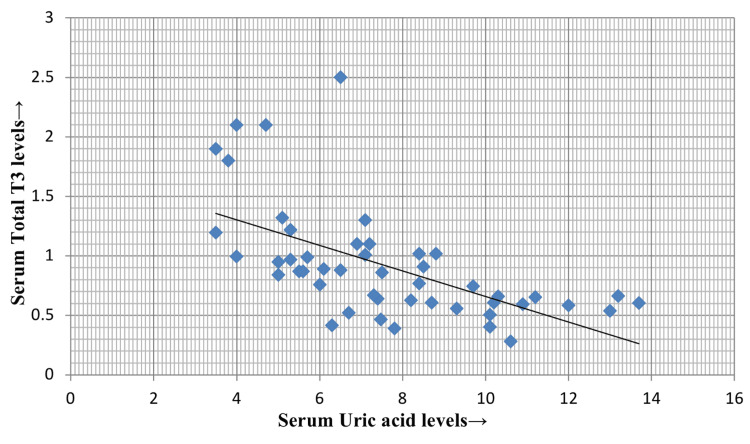
Correlation of serum uric acid with serum TT3 TT3: total T3

On the other hand, serum TT4 and TT3 were decreased in the study population, and both the parameters showed a negative correlation ((p = 0.001, r = -0.74) and (p = 0.001, r = -0.6)) with serum uric acid levels, as depicted in Figure 2 and Figure 3, respectively.

**Figure 4:**
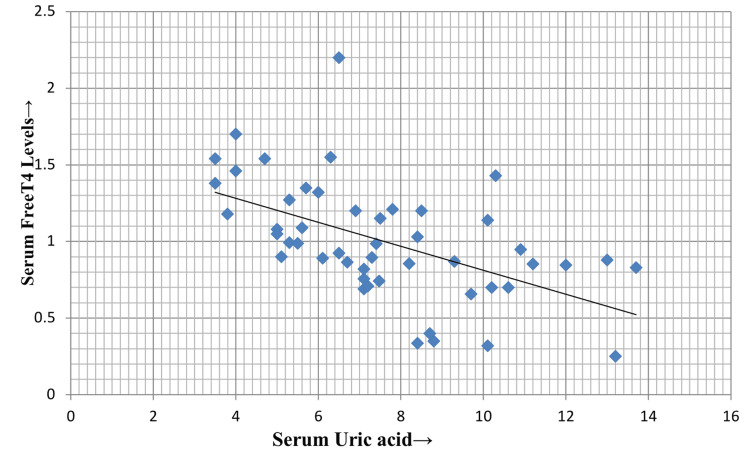
Correlation of serum uric acid with serum FT4 FT4: free T4

**Figure 5:**
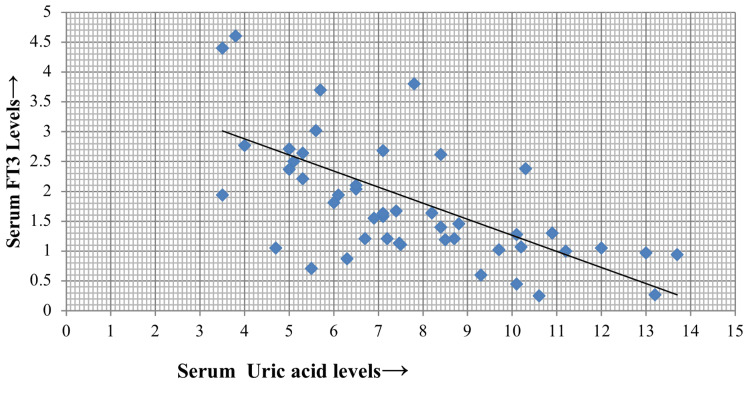
Correlation of serum uric acid with serum FT3 FT3: free T3

As depicted in Figure 4 and Figure 5, serum uric acid levels also showed a negative correlation with both FT4 (p = 0.001, r = 0.53) and FT3 (p = 0.001, r = 0.58), respectively.

## Discussion

The significant role of thyroid hormones in cellular growth, proliferation, and protein synthesis, as well as their association with the rapid development of renal tissues in neonatal rats, is well-documented [1,8,9]. There is a co-existence of CKDs and thyroid diseases with common etiological factors [10]. Previous studies have shown conflicting results regarding the association between serum uric acid levels and thyroid function in patients with CKDs. In our study, CKD patients exhibited significantly high levels of serum uric acid and serum TSH, with a positive correlation between both parameters. These levels also showed a positive correlation with serum creatinine and urea. However, in CKD patients, serum levels of TT4, TT3, FT4, and FT3 were decreased, and each of these parameters exhibited a negative correlation with serum uric acid levels.

Several studies have indicated that hyperthyroidism can lead to CKD by causing intra-glomerular hypertension due to increased filtration pressure, leading to hyperfiltration. Hyperthyroidism may also increase the risk of albuminuria, resulting in direct renal injury. In addition, it can induce increased mitochondrial energy metabolism and downregulation of superoxide dismutase, leading to increased free radical generation and renal injury. Oxidative stress in hyperthyroidism can accelerate hypertension, further contributing to the progression of CKD [11,12,13]. Hyperthyroidism is also linked to anemia in CKD patients and can cause resistance to recombinant human erythropoietin [14]. Previous studies have shown a strong association between hypothyroidism and CKDs [15,16,17]. Thyroid hormone abnormalities, such as reduced levels of TT3, TT4, FT3, and FT4, have been reported even in euthyroid CKD patients [18,19,20].

Some studies reported a high prevalence of elevated serum uric acid in hypothyroidism and hyperthyroidism [20,21,22]. This may be attributed to an elevated rate of purine metabolism in primary hyperthyroidism and reduced renal perfusion and glomerular filtration rate (GFR) in primary hypothyroid patients [23,24].

Serum uric acid is primarily excreted by the kidneys. In the presence of renal insufficiency, there is compensatory increased removal of uric acid by the gut, which is not entirely effective. As a result, serum uric acid levels increase as the GFR decreases, with approximately half of the subjects developing hyperuricemia by the time dialysis is initiated [22,25]. This complicates the assessment of uric acid's role in the progression of renal disease in individuals with CKD through epidemiological studies.

Several studies have also noted a significant increase in hypothyroidism in both urea and creatinine levels compared to hyperthyroidism [25,26]. Microscopic examination has revealed glomerular basement membrane thickening in nephrons of hypothyroid rats and humans. These changes can alter renal hemodynamics and reduce renal blood flow and GFR, consequently leading to reduced clearance of creatinine and uric acid [27]. Another possible mechanism of the above findings may be due to albuminuria in CKD patients, which results in hypothyroidism and ultimately the level of thyroglobulin increases, which carries T3 and T4 [28].

It is notable that while our study showed a correlation between serum uric acid level and thyroid function in CKD patients, a limitation was the lack of pre-disease evaluation. This makes it challenging to decide if CKD is a result of hyperuricemia with thyroid dysfunction or if CKD leads to hyperuricemia with abnormal thyroid function. Conducting a prospective experimental animal study or including a diverse range of healthy controls of the same ethnicity could offer valuable insights in this regard.

## Conclusions

In this study, there was a noteworthy and significant positive correlation between uric acid levels and TSH levels in patients with CKD. This finding suggests that as uric acid levels increase, TSH levels also tend to rise, indicating a potential interplay between these two biomarkers. Understanding this relationship is crucial, as it highlights the importance of monitoring serum thyroid profiles along with uric acid levels in CKD patients. Regular evaluation of these parameters could lead to earlier detection of complications associated with CKD. Furthermore, managing uric acid and thyroid hormone levels might improve patient outcomes and overall healthcare strategies in CKD management. Thus, incorporating thyroid and uric acid assessments into standard care practices for CKD patients could prove beneficial.
